# Supporting Sexual Health for People Living with Dementia: E‐Learning Modules to Build Confidence in Care Teams

**DOI:** 10.1002/alz70858_102829

**Published:** 2025-12-25

**Authors:** Katelynn Aelick, Kristy McKibbon, Rosemarie Mangiardi, Birgit Pianosi, Lori Schindel‐Martin

**Affiliations:** ^1^ Behavioural Supports Ontario Provincial Coordinating Office, North Bay, ON, Canada; ^2^ Hamilton Health Sciences, Hamilton, ON, Canada; ^3^ Ontario Health, London, ON, Canada; ^4^ Laurentian University, Sudbury, ON, Canada; ^5^ Toronto Metropolitan University, Toronto, ON, Canada

## Abstract

**Background:**

Behavioural Supports Ontario (BSO) provides behavioural healthcare services for older adults in Ontario with responsive behaviours associated with dementia, complex mental health, substance use, and/or neurological conditions. While BSO teams have developed expertise in assessing responsive behaviours and creating comprehensive care plans, challenges remain in addressing sexual health needs. Recognizing this knowledge gap, the Sexual Expression and Dementia (SED) Working Group developed a free, 3‐part e‐learning program designed to equip healthcare providers (HCPs) working in long‐term care (LTC) homes with best practice tools and resources to support the sexual health of residents with dementia.

**Methods:**

The e‐learning program was informed by an extensive literature review of over 300 articles which were supplemented by grey literature, case studies, and consultations with HCPs and field experts. This evidence‐informed process informed the development of the program's content as well as the case scenarios.

**Results:**

The e‐learning program was released in February 2025 on the AlzEducate learning management system. The first module shapes learners’ values, knowledge, and beliefs around sexual health and dementia. The second module provides HCPs with practical skills and tools for cultivating conversations about sexual health with LTC residents in a manner that prioritizes dignity and respect. The third module enables learners to evaluate the risk of sexual behaviours, and create care plans that balance safety and dignity. The program also offers downloadable resources that learners can incorporate into their practice to facilitate conversations about sexual health with individuals living with dementia, their care partners, as well as for assessment and documentation purposes.

**Conclusion:**

The *Supporting the Sexual Health of People with Dementia in LTC* e‐learning program offers accessible education on a topic vital to resident wellbeing, yet often overlooked. By equipping learners with practical tools, the program empowers HCPs to support the sexual health of people with dementia in their interactions with residents, care partners, and care teams. Evaluation of the series, using pre‐ and post‐surveys to assess changes in learner confidence and overall satisfaction, will inform the development of additional resources and modules to further support sexual health in dementia care.

